# Recent Views of Leprosy

**Published:** 1890-07-26

**Authors:** 


### THE PRACTITIONER'S MIRROR AND RETROSPECT.

[*The Editor will be glad to receive offers of co-operation and contributions from members of the profession. There is no doubt that much valuable material full of interest and instruction is at present lost to science. This is largely so in the case of* (1) *the younger and less-known members of the hospital staff* (2) *those connected with cottage hospitals, and* (3) *the great body of medical practitioners not attached to any institution. The co-operation of all is cordially invited to enable the Editor to make* THE HOSPITAL *of the utmost possible use and value to the profession. Specialists willing to review books on their special subjects are invited to communicate this fact at once. All letters should be addressed to* THE EDITOR, THE LODGE, PORCHESTER SQUARE, LONDON, W.]

### MEDICINE.

### RECENT VIEWS OF LEPROSY.

We have several times referred to the subject of leprosy, and now do so once again; especially as Mr. Hutchinson has quite recently published interesting papers on the disease. Mr. Hutchinson says there is no evidence in support of the belief that leprosy ever has a primary lesion, or that its transference is effected by means of a sore on the external parts. There is nothing, he says, which corresponds to the sores of specific disease, or to the ulcer of malignant pustule. This, however, can scarcely be regarded as conclusive proof that the system may not be contaminated without any sore being produced at the point of entrance of the poison. When a person is bitten by a rabid dog the injured part heals, and often, although the person may suffer from hydrophobia, the bitten part is not further affected. Moreover, instances have occurred of the direct communication of leprosy from one person to another. There is the experiment, quite recently made, on a criminal at Honolulu. This criminal was inoculated with leprous discharge in 1885, and was found to be a leper in 1888. There is also a case of a boy named Miller, who pricked himself with a needle which had been used by a leper, and became affected with the disease. A case was also recently reported from Bombay of a medical student injuring himself when engaged on a post-mortem examination of a leper, and becoming afflicted. Of course it may be objected that environment may have had some influence in the production of leprosy in these persons. And this is quite possible. But if we recollect rightly, Professors Damisch and Kobner proved that leprosy may be communicated to animals by inoculation. If, as is now asserted, leprosy depends upon a microbe or bacillus, there does not appear any reason why such bacillus should not be conveyed directly from one person to another. Popular belief in all countries, and at all times, has pointed to the propagation of leprosy by contagion. In tropical countries, the spread of the disease by this means appears easy, and it is in tropical countries that there is the intensity of leprosy. The people do not wear, as a rule, either shoes or stockings. Usually they wear slippers, which are frequently put off when entering a dwelling. Owing to the habit of walking bare-foot, or with only slippers, feet are often scratched and wounded. By mistake, a leper's slippers may be used, and leprous discharge may be conveyed to the sore foot of a healthy person. Notwithstanding the very able arguments of Mr. Hutchinson, we cannot accept the proposition that leprosy is not contagious, although, as is the case with other maladies, the disease may be, and perhaps usually is, conveyed in other ways. Speaking of the manifestation of leprosy by the appearance of dusky patches on the skin, Mr. Hutchinson observes that such patches are not the initial lesion, but are due to implantation of the bacilli from the blood, and with this we fully agree. Treatment of such patches is useless, for they are simply the local manifestations of a constitutional disorder.

Mr. Hutchinson states that the malady is one and the same everywhere, and that the several terms in use, such as macular, tuberculous, anæsthetic, &c., have reference, not to true varieties, but to different phenomena in the same case. This, again, is, according to our experience, quite correct; but we cannot agree with Mr. Hutchinson when he observes, "No case of leprosy ever begins by anæsthesia, or by so-called tubercles." Now, we believe that leprosy very often begins by anæsthesia, generally of some part of the foot or leg. This symptom is well known to the natives of India, under the term *sun*, and *sun* may be complained of when no other symptom of leprosy can possibly be detected; before there are any patches on the skin, or tubercles of the skin. No doubt, as Mr. Hutchinson seems to think, the term tubercle is a misnomer for the formations seen in connection with leprosy; but the term has come into use, and will not easily be discarded. A tubercular or pendulous condition of the ear-lobes is sometimes one of the very early signs of leprosy. Other early signs are a deviation of the palatine arches, probably from anæsthesia; a thickness of speech; affections of the gums resembling scurvy; anæthesia of the little finger; atrophy of the ball of the thumb; chronic conjunctivitis, languor, depression, deep-seated rheumatic pains. One or several of the above conditions may be evident long before any decided symptoms of the disease make their appearance. It has been stated on good authority that leprosy may be diagnosed in the earlier stages by taking the patient into a dark room and passing a blue flame before the face. If the disease is present the skin assumes a reddish hue. If there is a greenish hue there is no leprosy. This test is said to be used by the Chinese, but having been tried in India did not succeed.

Mr. Hutchinson has several times sought to connect leprosy with fish eating. And there is no reason why the bacillus of leprosy may not inhabit a fish, and so be conveyed with a fish diet into the human system. It is well known that various kinds of fish, especially in the tropics, harbour different kinds of living atoms. The subject was some years back thoroughly investigated by the late Deputy Surgeon-General Furnell, of Madras. Dr. Furnell described a large number of parasites found in fish, but saw no reason to connect them with either cholera or leprosy, although he did connect them with "worms." Then there is the fact of leprosy prevailing to a considerable extent among inhabitants of coast districts, who naturally consume fish as a principal article of diet. But, unfortunately for the fish theory, leprosy prevails, in India for instance, to almost the same extent in many parts of the interior of the country, hundreds of miles from the coast, where the people never see sea-fish, and very rarely fresh-water fish. In some parts of Western India water is three or four hundred feet from the surface. A little pool, if perchance anything of the kind exists, is honoured by the title of a "great big lake." Fish is an unknown commodity. Yet leprosy is there. Morever, the Bunina class, who live on the coasts as elsewhere—and who never eat fish—are not exempt from leprosy. As remarked above, it is quite possible that leprosy bacillis may be conveyed by fish, but that leprosy is generally consequent on a fish diet, is not in accordance with facts. Several other causes have been mentioned with equally good reason. It has been supposed to be due to a want of salt in the diet. Doubtless salt is necessary both for human beings and for animals. It is a known fact that the hair of oxen deprived of salt becomes matted, rough, and falls off. But leprosy prevails in districts where there is no lack of salt—throughout the salt-producing centres of Western India, for instance. Leprosyhas been attributable to an exclusively vegetable diet, but it prevails among those who eat meat. The kind of food used does not appear to influence the disease, unless from insufficiency producing a state of system "below par," which however favours the invasion of most diseases.

Dr. Ransome has had something to say quite recently about leprosy, in his lecture on "The Etiology and Prevention of Phthisis." He points out that there are certain features of resemblance between the two diseases. He asserts, that the nearest approach to tubercle is to be found in leprosy. A micro-organism is closely associated with each disease. Leprosy is like tubercle in its mode of attack. Both maladies are slow in progress. The pathology of the two diseases is strikingly similar. Both are distinctly endemic. Precisely the same controversy now waged with regard to phthisis, is carried on as to the contagiousness of leprosy. Both have been attributed to heredity. It is not surprising, therefore, that the question should be asked, Is not leprosy a tubercular disease? There is, however, a great difference in the statements made by bacteriologists as regards the behaviour of the two bacilli. The leprosy bacilli are reputed to have the power of infecting the nerves, or rather the sheaths of the nerves, without infecting necessarily other parts of the body. And in this manner it is sought to explain the anæsthesia of leprosy. But tubercle bacilli do not appear to have any special preference for nervous tissue. The lungs and viscera generally are the parts in which those bacilli flourish best. There are, however, many other diseases which have a striking similarity; the different forms of one fever, for instance; croup and diphtheria; bad cases of diarrhœa and cholera. But the points in which leprosy does *not* agree with tubercular disease are much more prominent than the items of similarity. The symptoms, some of which have been noticed above, are not those of tubercular disease, and the results are not those of tubercular disease, for they more nearly resemble the ravages of specific disease. Recently (*British Medical Journal*) Sir William Moore has brought forward various cogent reasons why he regards leprosy as a manifestation of hereditary specific disease. The fact is, that although so much has been written on the subject of leprosy, we have still much to learn. The matter is one which should no longer be left to individual enterprise. It is a subject which the State should take up. Perhaps it may be effectually investigated under the auspices of the committee over which His Royal Highness the Prince of Wales presides. We have not, however, heard that anyone, as proposed, has been nominated to undertake the labour of investigating leprosy. If sufficient funds are not forthcoming from the public, the expense should be borne by the State; for Imperial responsibilities are involved. In the meantime we may well rest from our efforts to establish theories which may probably prove but conjecture and speculation.

